# Sonochemical Synthesis of Copper-doped BiVO_4_/g-C_3_N_4_ Nanocomposite Materials for Photocatalytic Degradation of Bisphenol A under Simulated Sunlight Irradiation

**DOI:** 10.3390/nano10030498

**Published:** 2020-03-10

**Authors:** Gang-Juan Lee, Xin-Yu Lee, Cong Lyu, Na Liu, Sambandam Andandan, Jerry J. Wu

**Affiliations:** 1 Department of Environmental Engineering and Science, Feng Chia University, Taichung 407, Taiwan; leeganjiuan@gmail.com (G.-J.L.); chatter850603@gmail.com (X.-Y.L.); 2 College of New Energy and Environment, Jilin University, Changchun 130021, China; lvcong@jlu.edu.cn (C.L.); liuna@jlu.edu.cn (N.L.); 3 Nanomaterials & Solar Energy Conversion Lab, Department of Chemistry, National Institute of Technology, Trichy 620015, India

**Keywords:** sonochemical synthesis, composite nanomaterials, photocatalytic degradation

## Abstract

Copper-doped bismuth vanadate/graphitic carbon nitride (BiVO_4_/g-C_3_N_4_) nanocomposite materials were successfully fabricated using a sonochemical approach. Cu-doped BiVO_4_/g-C_3_N_4_ nanocomposite photocatalysts could improve electron/hole (e^−^/h^+^) pair separation, stability, and light-harvesting efficiency compared to pristine BiVO_4_ or g-C_3_N_4_, resulting in the enhancement of photocatalytic activity. The optimal parameters, such as pH value at 10, photocatalyst dosage of 0.4 g L^−1^, and 10 mol% Cu-doped BiVO_4_/g-C_3_N_4_ photocatalyst, were determined to degrade initial concentration of 20 ppm Bisphenol A, which could be completely removed after 90 min. Furthermore, the excessive doping of copper (> 10 mol%) could not synthesize the pure monoclinic scheelite phase, which substantially resulted in the reduction of the photocatalytic activity.

## 1. Introduction

Bismuth vanadate (BiVO_4_) has three crystal phases, such as tetragonal-scheelite, monoclinic scheelite, and zircon. Among those, the monoclinic phase, in particular, exhibits outstanding visible-light photocatalytic properties because monoclinic scheelite BiVO_4_ possesses the small bandgap 2.4 eV [1,2,3,4,5]. Dong et al. (2016) [6] prepared monoclinic scheelite BiVO_4_ semiconductors with porous structures using a two-step method. The porous BiVO_4_ material prepared at hydrothermal temperatures of 200 °C displays enhanced photocatalytic efficiency by degrading methylene blue (MB). Sun et al. (2019) revealed that the two-dimensional BiVO_4_ nanosheets/reduced graphene oxide (RGO) exhibited higher photocatalytic activity [7]. In addition, the heterojunction of semiconductor composite has been reported to be an effective strategy to fabricate efficient photocatalysts [6]. Xie et al. (2018) exhibited that the BiVO_4_/Mn-Zn ferrite (Mn_1−x_Zn_x_Fe_2_O_4_)/RGO photocatalyst could degrade rhodamine B (RhB) about 96% [8]. Thus, in this article, we chose graphitic carbon nitride (g-C_3_N_4_) to modify BiVO_4_ to enhance photocatalytic efficiency due to graphene-like structure, good thermal and chemical stability, good visible light absorption, and photocatalytic properties of g-C_3_N_4_ [6]. In addition, g-C_3_N_4_ nanosheets could enhance an easier electron transfer during the reaction [9]. Smykalova et al. (2019) indicated that g-C_3_N_4_ nanosheets possessed a large specific surface area, which resulted in the higher degradation ratio of medicines in water [10]. Zhang et al. (2017) indicated that the composite with 7 wt% g-C_3_N_4_ had better photocatalytic efficiency than bare BiVO_4_ [11]. The stable core/shell structure of the BiVO_4_/g-C_3_N_4_ composite material not only enlarges the surface area but also enhances the photo-generated charge separation. Therefore, a new composite photocatalyst is expected to improve the stability, solar light utilization, charge separation, and transfer by combining with the two semiconductors.

BPA (bisphenol A) is not only a synthetic organic chemical with various applications in the polymer industry used as intermediate for the production of epoxy resins and plastics but also an endocrine disruptor, which would lead to serious ecology exacerbation [12,13]. Some researchers have shown that exposure to very low BPA levels may result in reduced fertility and increased incidence of breast, ovarian, and testicular cancers [13]. Therefore, BPA should be completely removed from the water for safety consideration to human beings. In this research, we prepared the BiVO_4_ nanocomposite photocatalysts at 60 °C, 1 atm, and 1.5 h via a sonochemical synthesis way. The advantage of sonochemistry in fabricating nanostructured samples occurs mostly from acoustic cavitation [14]. In addition, we also introduced the synthesis of copper and g-C_3_N_4_-modified BiVO_4_ and removal of BPA using photocatalytic reaction under simulated sunlight irradiation. Some parameters that affect the efficiency of BPA removal via the photocatalytic degradation process, including the pH values in solution, the dosages of BiVO_4_ nanocomposite material, and the various BiVO_4_ nanocomposite materials, were studied.

## 2. Methods 

### 2.1. Sonochemical Synthesis of BiVO_4_ Nanocomposite Photocatalysts

A total of 5 g urea (NH_2_CONH_2_, NIHON SHIYAKU REAGENT) was added into an aluminum crucible, and subsequent thermal treatment at 550 °C for 2 h with 5 °C min^−1^ of heating rate in argon atmosphere could obtain a light-yellow powder composed of g-C_3_N_4_ nanoparticles. A total of 3.38 g bismuth (III) nitrate pentahydrate (Bi(NO_3_)_3_·5H_2_O, Alfa Aesar) was placed in 40 mL of 2 M nitric acid solution (solution A). Then, 0-40 wt% g-C_3_N_4_ was put into solution A to be stirred for 1 h. The 0.81 g ammonium metavanadate (NH_4_VO_3_, Acros) was dissolved in 40 mL of deionized (DI) water (solution B). The 1 wt% surfactant, such as citric acid monohydrate (CIT, C_6_H_8_O_7_•H_2_O, fw: 192.12, SHOWA), (1-hexadecyl)trimethylammonium bromide (CTAB, CH_3_(CH_2_)_15_N(CH_3_)_3_Br, fw: 364.42, Alfa Aesar), and polyethylene glycol (PEG, C_2n_H_4n+2_O_n+1_, fw: 4,000, SHOWA), was added into the solution B. The 0-20 mol% copper (II) acetate monohydrate (Cu(CH_3_COO)_2_•H_2_O, Merck) was also added in 20 mL of DI water (solution C). Subsequently, solution A and solution C were added into solution B. Then, the reaction mixture was treated with a sonochemical instrument (700 W, 20 kHz, Q700 SONICATOR) for 0.5 h–2 h at 60 °C [4]. Finally, the copper-doped BiVO_4_/g-C_3_N_4_ samples were collected.

### 2.2. Characterization of BiVO_4_ Nanocomposite Photocatalysts

The morphologies were examined by the JEOL JSM-7800F model and the JEOL JEM-2010 model. The XRD patterns were examined by the Rigaku Ultima III diffractometer (Rigaku Japan Sales Division, Tokyo, Japan). Particle size distribution, bandgap, and photoluminescence (PL) properties were measured using the Shimadzu SALD-2300 model, Shimadzu UV-2600 and Shimadzu RF-3501 spectrometer (Shimadzu Corporation, Tokyo, Japan), respectively. The flat-band potential of the sample was recorded using potentiostat/galvanostat PGSTAT302N, Metrohm Autolab (Metrohm AG, Herisau, Switzerland). The surface area, pore size, and pore volume were measured by using a Micrometrics ASAP-2020 nitrogen adsorption instrument (Micrometrics Headquarters, Norcross, GA, USA).

### 2.3. Photocatalytic Reaction

Typically, an appropriate amount of BiVO_4_ nanocomposite material was put into 100 mL of BPA solution (4,4’-dihydroxy-2,2-diphenylpropane, Bisphenol A, C_15_H_16_O_2_, ECHO) with a concentration of 20 ppm. The reactor was irradiated with a 350 W Xenon light (KIT-XENON-ADJ350W) for 6 h. During the experiment, about 1.5 mL sample was withdrawn at predetermined time intervals, and the sample was immediately filtered through the 0.22 μm polyvinylidene difluoride (PVDF) syringe filter to remove the powders, and the clarified solution was analyzed by High-performance liquid chromatography (HPLC, LC-20A, Shimadzu Corporation, Tokyo, Japan). 

## 3. Results

### 3.1. Characterization of BiVO_4_ Nanocomposite Photocatalysts

**Figure 1:**
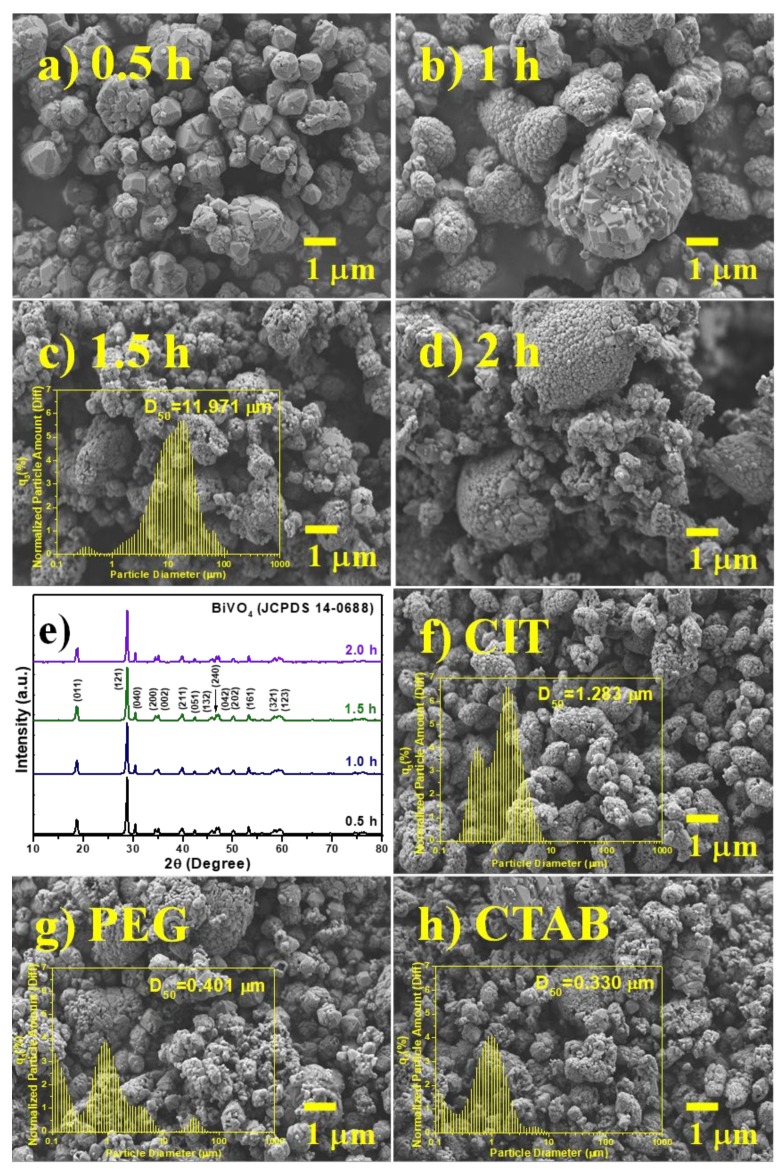
FESEM images (**a**–**d**), and XRD patterns (**e**) of BiVO_4_ photocatalysts with different ultrasound irradiation time (without surfactant). FESEM images (**f**–**h**) of BiVO_4_ materials via various surfactants at 1 wt%. Insets reveal the particle size distribution for the corresponding samples.

Reaction conditions could effectively affect the morphology of photocatalysts. Figure 1a–d show when irradiation time was extended, the particle size of BiVO_4_ nanoparticles could gradually enlarge. As time was irradiated for 1.5 h, BiVO_4_ samples had the uniform particle size and the rough surface. The phase and crystallographic nanostructures of the BiVO_4_ particles are shown in Figure 1e. The major diffraction peaks at 18.83°, 28.84°, 30.56°, 34.51°, 35.22°, 39.96°, 42.40°, 45.84°, 46.74°, 47.25°, 50.28°, and 53.22° belonged to the (011), (121), (040), (200), (002), (211), (051), (132), (240), (042), (202), and (161) planes of BiVO_4_ nanostructures and matched well with the crystal phase of monoclinic BiVO_4_ (JCPDS Card No. 14-0688, cell parameter a = 5.195 Å, b = 11.70 Å, and c = 5.092 Å). No other impurities could be detected. This also indicated that the ultrasonic time did not influence the crystal structure. According to our previous study, surfactant would affect the formation of various morphological structures [15]. Therefore, we used three types of surfactants to control the particle size of BiVO_4_, such as CIT (chelating agent), CTAB (cationic surfactant), and PEG (non-ionic surfactant). Figure 1f–h show FE-SEM images and particle size distribution of BiVO_4_ with various surfactants. The particle size distribution (D_50_) of BiVO_4_ with surfactants significantly decreased from 11.971 μm to 0.330 μm, which could be attributed to better dispersibility by adding the surfactants with long molecular chain and hydrophilic end during the synthetic process [16,17,18]. Therefore, the smaller particle size could be formed than that of pristine BiVO_4_.

**Figure 2:**
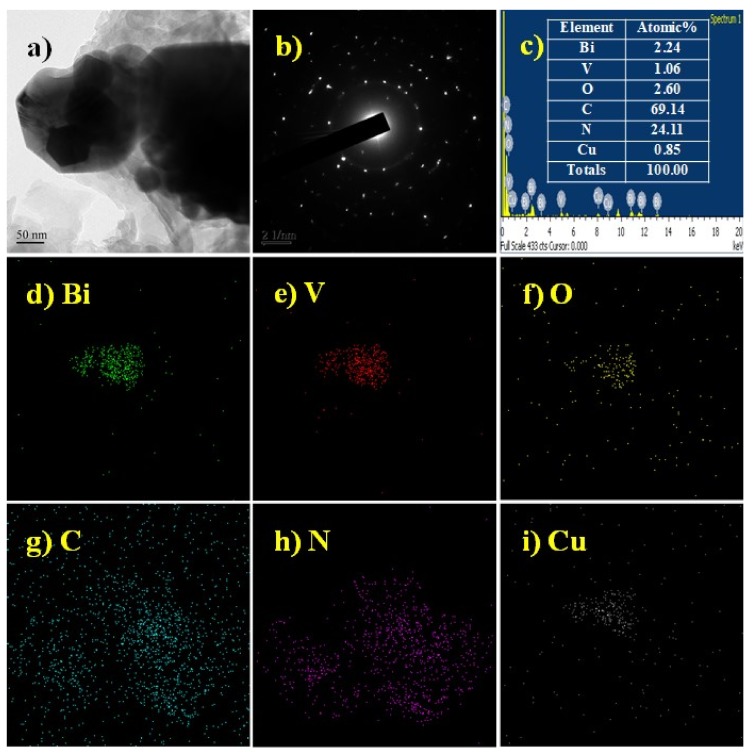
Typical TEM image (**a**), corresponding selected area electron diffraction (SAED) pattern (**b**), EDX (**c**), and elemental mapping record Bi, V, O, C, N, and Cu (**d**–**i**) of the 10Cu/BiVO_4_/g-C_3_N_4._

**Figure 3:**
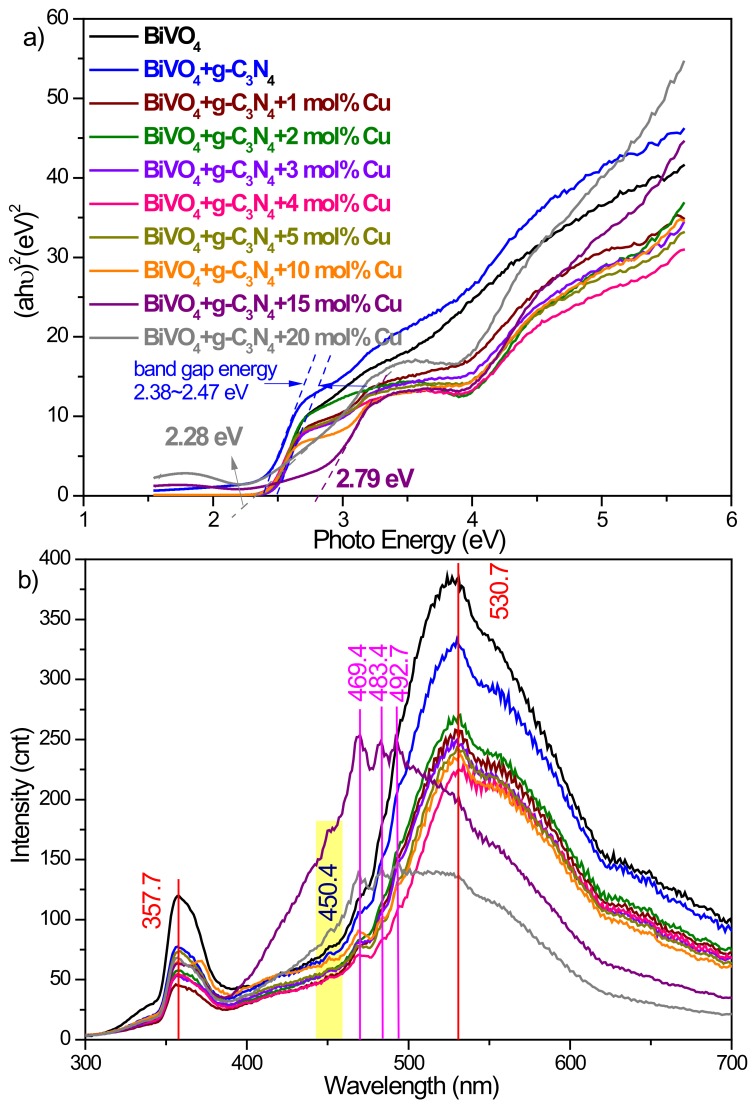
(**a**) UV-Vis absorption spectra, and (**b**) photoluminescence spectra of BiVO_4_/g-C_3_N_4_ nanocomposite materials with various amounts of copper.

**Figure 4:**
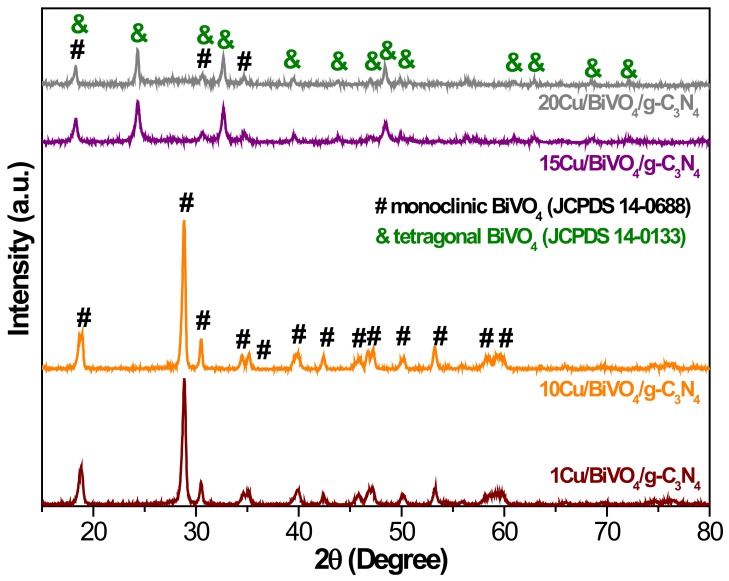
XRD patterns of BiVO_4_/g-C_3_N_4_ nanocomposite materials with various amounts of copper.

The BiVO_4_ photocatalyst was further modified by g-C_3_N_4_ and copper in order to promote the photocatalytic activity. The results were confirmed by HR-TEM measurement on the 10Cu/BiVO_4_/g-C_3_N_4_ powders (Figure 2). The corresponding selected area electron diffraction (SAED) pattern indicated rings; the as-synthesized BiVO_4_ nanocomposite photocatalysts was the polycrystalline structure (Figure 2b). Furthermore, the element of Bi, V, O, C, N, and Cu substantially existed in the sample from the EDX image and TEM-EDX element mapping (Figure 2c–i). The bandgap of g-C_3_N_4_ and copper-modified BiVO_4_ slightly reduced from 2.47 eV to 2.28 eV (Figure 3a). The PL spectra of the BiVO_4_ samples are exhibited in Figure 3b. BiVO_4_ photocatalyst displayed two peaks at 530.7 nm and 357.7 nm [19]. The g-C_3_N_4_ had its characteristic peak around at 450.4 nm [20]. In addition, the pristine BiVO_4_ sample had higher PL intensity than that of g-C_3_N_4_ and copper-modified BiVO_4_ samples. In other words, the e^−^/h^+^ recombination rate could be efficiently restrained in the g-C_3_N_4_ and Cu-modified BiVO_4_ samples. In the high concentration of copper (> 10 mol%), the PL peak of BiVO_4_ nanocomposite photocatalysts shifted from 530.7 nm to 492.7–469.4 nm due to the fabrication of the tetragonal-scheelite structure BiVO_4_, as shown in Figure 4. Therefore, the excessive doping of copper (> 10 mol%) could not synthesize the pure monoclinic scheelite phase, which might result in the reduction of the photocatalytic activity.

### 3.2. Photocatalytic Degradation Activity

The photocatalytic degradation activities of the BiVO_4_ nanocomposite photocatalysts were performed for the degradation of wastewater pollutants containing BPA under simulated sunlight irradiation. Photocatalytic parameters of degrading BPA were investigated as below, including the pH values of BPA solution, the dosages of BiVO_4_ nanocomposite photocatalyst, and the content of copper.

#### 3.2.1. Initial pH of the BPA Solution

**Figure 5:**
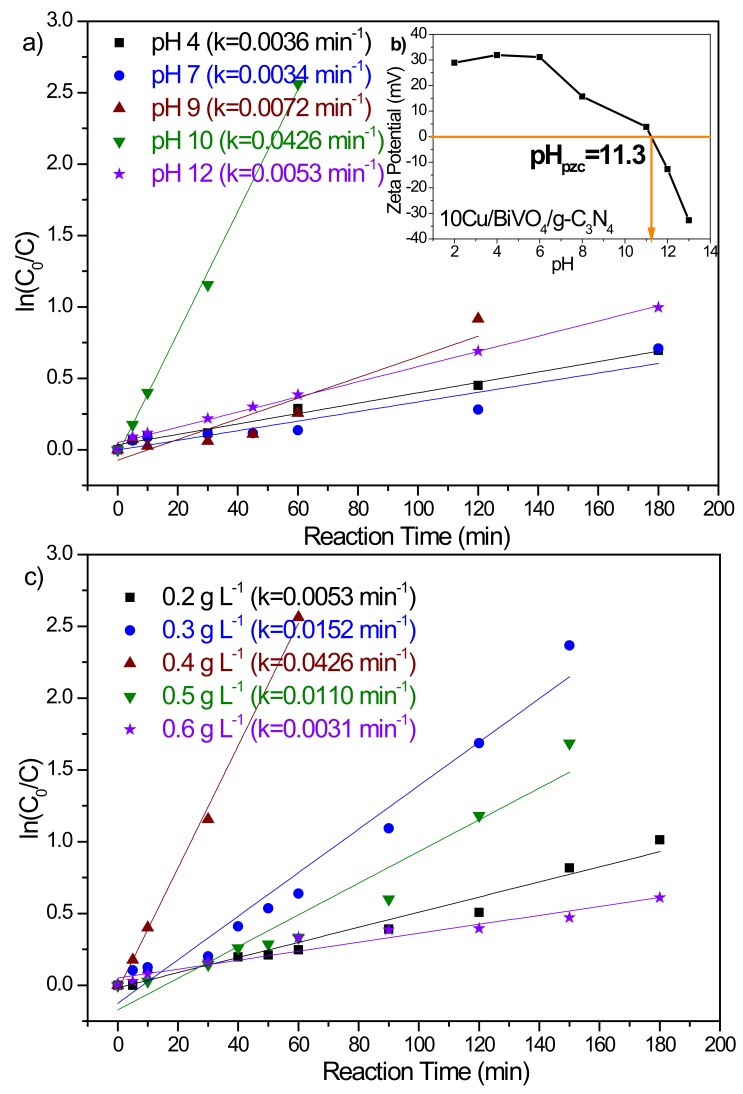
(**a**) Effect of bisphenol A (BPA) photodegradation at the different initial solution pH values on 10Cu/BiVO_4_/g-C_3_N_4_ photocatalyst. ((BPA): 20 mg L^−1^; catalyst concentration of 0.4 g L^−1^), (**b**) The zeta potential values of 10Cu/BiVO_4_/g-C_3_N_4_ photocatalyst, (**c**) Changes of the apparent BPA photodegradation reaction kinetics at a different dosage of 10Cu/BiVO_4_/g-C_3_N_4_ photocatalyst. ((BPA): 20 mg L^−1^; pH = 10).

Figure 5a plots the photo-degradation of BPA at various pH values. The results could be well fitted by pseudo-first-order kinetics model with a rate constant of 0.0036 min^−1^, 0.0034 min^−1^, 0.0072 min^−1^, 0.0426 min^−1^, and 0.0053 min^−1^ for pH value of 4, 7, 9, 10, and 12, respectively. With the increase of the pH value to 10, the BPA could be completely removed to 100% for 120 min. This result could be illustrated by the net signs of the surface charge of the BPA species and BiVO_4_ nanocomposite photocatalysts at different pH values. Because the dissociation constants (pK_a1_ and pK_a2_) of BPA are 9.6 and 10.2 [21,22,23], the surface charge of the BPA species is negatively charged under alkaline conditions. In addition, the pH changes also could affect the surface properties of 10Cu/BiVO_4_/g-C_3_N_4_ photocatalyst. Meanwhile, we measured the point of zero charge of 10Cu/BiVO_4_/g-C_3_N_4_ photocatalyst (pH_pzc_ = 11.3). Therefore, BPA molecules were adsorbed onto the surface of 10Cu/BiVO_4_/g-C_3_N_4_ photocatalyst via electrostatic interaction (pH smaller than 11.3), which is an important step in the process of photocatalytic degradation. Conversely, BPA was adsorbed weakly onto the negatively charged 10Cu/BiVO_4_/g-C_3_N_4_ surfaces due to the coulombic repulsion (pH greater than 11.3). Based on the above results, the best pH value of the BPA solution for the photocatalytic degradation of BPA was at 10.

#### 3.2.2. Photocatalyst Dosage

The photocatalytic efficiency by using metal oxides can be determined by their physical and chemical properties. Physical properties, such as pore size, surface area, and surface charge, could affect the photocatalytic activity [24]. Therefore, the function of the photocatalysts is able to provide effective surface area and play the role of the active center. The effect of photocatalyst dosage on the degradation efficiency was evaluated by various amounts of 10Cu/BiVO_4_/g-C_3_N_4_ from 0.2 g to 0.6 g in a 1000 mL BPA solution. The result of the photo-degradation performance has been depicted in Figure 5c, which exhibits that the rate of degradation increased linearly with an increase in the amount of photocatalyst up to 0.4 g and then decreased. The presence of 10Cu/BiVO_4_/g-C_3_N_4_ photocatalyst could provide more active centers, which led to increased photocatalytic activity. As the amount of photocatalyst increased, the number of BPA molecules adsorbed were increased, leading to an increase in BPA degradation. At higher concentrations of photocatalyst, more surface areas were available for constant BPA molecules. However, more photocatalyst would also induce greater aggregation of the photocatalyst, and the specific surface area decreased, leading to a reduction in the reaction rate. In addition, the inactivation of activated molecules by collision with ground-state molecules might also hinder the photocatalytic efficiency. Hence, above a certain level, additional catalyst amounts were not involved in catalysis reaction, and thus the rate might level off. Hence, the appropriate photocatalyst dosage showed an outstanding performance of photocatalytic degradation.

#### 3.2.3. Cu Doping Content

**Figure 6:**
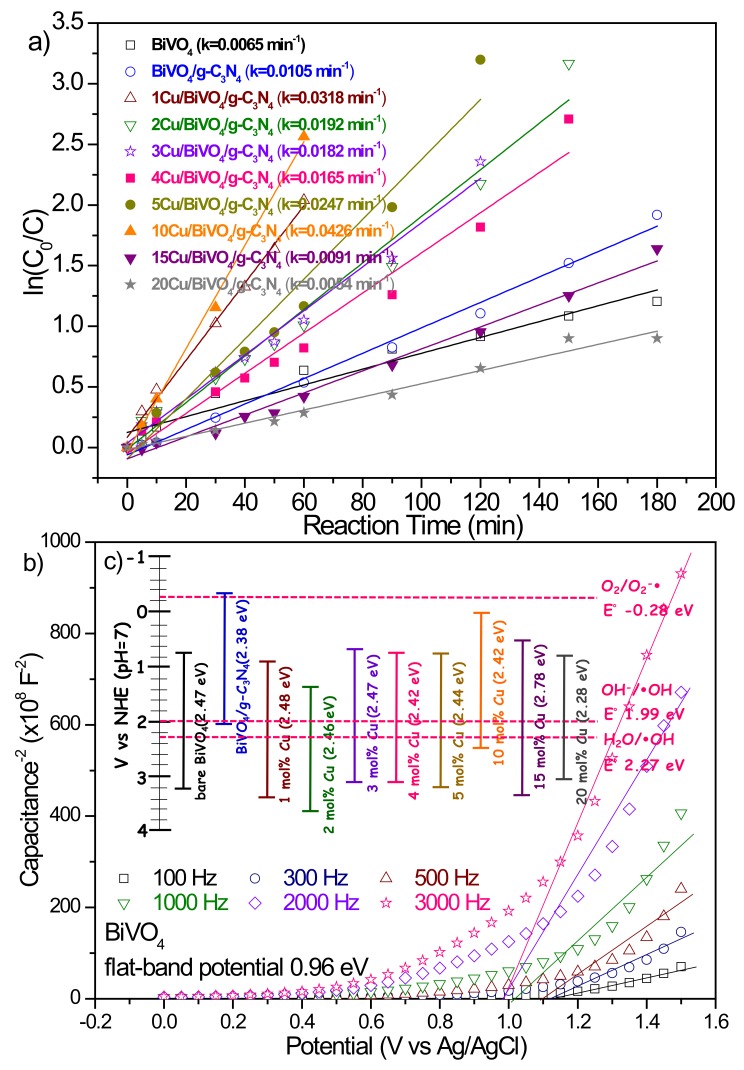
(**a**) Changes of the apparent BPA photodegradation reaction kinetics at different photocatalysts. ((BPA): 20 mg L^−1^; pH = 10; catalyst concentration of 0.4 g L^−1^), (**b**) Mott–Schottky plots for BiVO_4_, (**c**) Energy-level diagram displaying the conduction band and valence band edge positions of BiVO_4_/g-C_3_N_4_ nanocomposite photocatalysts with different amounts of copper.

**Figure 7:**
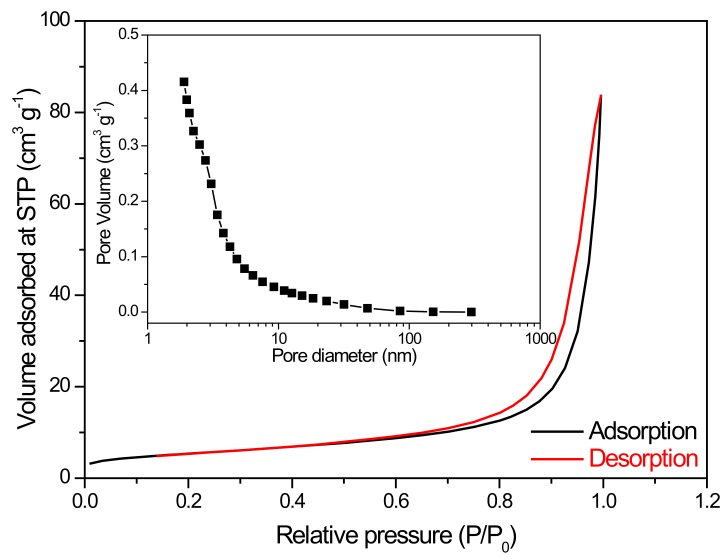
Nitrogen adsorption-desorption and Brunauer-Emmett-Teller (BJH) pore diameter distribution of 10Cu/BiVO_4_/g-C_3_N_4_.

In view of the above results, we conducted the experiments of the photocatalytic degradation of BPA by BiVO_4_ nanocomposite photocatalyst equal to 0.4 g L^−1^ at pH 10. Figure 6a exhibits the photocatalytic activity of BiVO_4_ nanocomposite photocatalysts with various Cu contents. It could be seen that the Cu content in the BiVO_4_ presented an excellent influence on the photocatalytic activity of BiVO_4_ nanocomposite materials. The conduction band (CB) edge position and valence band (VB) edge position of bare BiVO_4_ were about 0.75 eV and 3.22 eV, respectively, leading to a weak reduction ability, as shown in Figure 6b. The 10 mol% Cu-doped BiVO_4_ photocatalyst could not only shift the CB edge position more negative than the others but also decrease the intensity of PL emission (Figure 3b). Therefore, 10Cu/BiVO_4_/g-C_3_N_4_ photocatalyst had the highest activity (k = 0.0426 min^−1^) for photocatalytic degradation. Moreover, the addition of excess Cu contents (> 10 mol%) to synthesize BiVO_4_ nanocomposite photocatalysts resulted in a reduction in the photocatalytic activity because BiVO_4_ formed the tetragonal-scheelite phase, as shown in Figure 4. According to the references, we know that the crystal structure of monoclinic type BiVO_4_ exhibits excellent photocatalytic properties [2,3,4]. In addition, copper-doped photocatalyst can fabricate the trap energy levels (TEL), and the activated electrons could transfer to TEL_Cu_, which leads to the useful e^−^/h^+^ separation [15,16,25,26,27]. Therefore, the appropriate addition of Cu contents displayed an excellent photocatalytic degradation performance. Furthermore, the 10Cu/BiVO_4_/g-C_3_N_4_ was assigned to the type IV isotherm, displaying with a type H3 hysteresis loop, which indicated the presence of mesopores (2–50 nm), as shown in Figure 7. The BiVO_4_/g-C_3_N_4_ nanocomposite materials with various amounts of copper addition are compared in Table 1, revealing a slight decrease in the surface areas with copper doping. However, the slight loss of specific surface area did not compete with the enhanced activity of doping copper as reaction centers for photocatalytic degradation of BPA. 

## 4. Conclusions

BiVO_4_ nanocomposite photocatalysts were prepared using the sonochemical approach. Cu-doped BiVO_4_/g-C_3_N_4_ nanocomposite photocatalysts exhibited a remarkable improvement in degradation performance. The pH values of BPA solution, the dosages of 10Cu/BiVO_4_/g-C_3_N_4_, and the various types of BiVO_4_ nanocomposite material had a great influence on the removal efficiency of BPA. The optimal parameters, such as pH value at 10, photocatalyst dosage of 0.4 g L^−1^, and 10 mol% Cu-doped BiVO_4_/g-C_3_N_4_ photocatalyst, were determined to degrade the initial concentration of 20 ppm bisphenol A, which could be completely removed after 90 min.

## Figures and Tables

**Table 1:** The surface area of BiVO_4_/g-C_3_N_4_ nanocomposite materials with various amounts of copper.

Cu (mol%)	0	1	2	3	4	5	10	15	20
Surface area (m^2^/g)	23.1	22.6	22.1	21.5	20.6	20.0	19.4	17.0	15.6
